# CD4, CD8 and natural killer cells are depressed in patients with alopecia areata: their association with disease activity

**DOI:** 10.1186/s12865-022-00486-4

**Published:** 2022-03-17

**Authors:** Abdel-Khalek Younes, Refaat Hammad, Mona Othman, Ali Sobhy

**Affiliations:** 1 Dermatology, Andrology and STIs Department, Faculty of Medicine, Al-Azhar University, Assiut, Egypt; 2 Clinical Pathology Department, Faculty of Medicine, Al-Azhar University, Assiut, Egypt

**Keywords:** Alopecia areata, CD4 T cells, CD8 T cells, Natural killer cells

## Abstract

**Background:**

Alopecia areata (AA) is a common inflammatory disorder targeting the hair follicles leading to non-scaring hair loss. The pathogenesis of AA is still unclear, despite the accumulating evidence of the immune-mediated nature of this disease. So, in this study, we aimed to assess the level of CD4 T cells, CD8 T cells and natural killer (NK) cells in the peripheral blood of patients with active AA and control subjects, and to evaluate the association between the level of those cells and the duration of disease in different subtypes of AA.

**Results:**

Fifty female patients and 50 age- and sex-matched healthy controls were enrolled in this case control study. CBC analysis and the level of CD4, CD8 T cells and NK cells were evaluated during the active stage of the disease. We found that CD4, CD8 T cells and NK cells proportion was significantly lower (P < 0.05) in patients with active stage AA compared with healthy subjects, however, the ratio of CD4:CD8 T cells was significantly higher in patients than control subjects. The level of CD4, CD8 T cells CD56 bright CD16^−^ % NK cells were positively correlated with the disease duration.

**Conclusion:**

Active stage of AA disease is associated with a reduction of the circulating CD4, CD8 T cells and NK cells and an increase in CD4/CD8 T cells ratio, however, the level of those cells were higher with prolonged disease duration. Our findings confirm that immune mechanisms are involved in the pathogenesis of AA.

**Supplementary Information:**

The online version contains supplementary material available at 10.1186/s12865-022-00486-4.

## Introduction

AA is a widespread inflammatory autoimmune disease that attacks the hair follicles during the anagen phase [1]. It is characterized by non- cicatricial hair loss affecting about 2% of the population of both sexes and all age groups [2, 3]. Clinically, patients can be presented with patchy, diffuse, or total body hair loss [2, 3].The pathogenesis of alopecia areata is still unclear, however, there is accumulating evidence hypothesized that alternation in CD4, CD8 T cells and their cytokines are the main inducers of hair loss [4].

Immunohistologically, AA is associated with the disturbance of the numbers and function of peri-and intra-follicular inflammatory cells, and this disturbance is more abundant during the active stage of the disease [5]. CD8 + T cells attack the intra-follicular regions, while CD4 + T cells and NK cells are accumulated around the outer root sheath of hair follicles [6]. This imbalance in the inflammatory cells, leading to a collapse of the immune privilege of the hair follicle [7, 8].

The disturbance of the inflammatory cells is not only restricted on the site of the lesion, but it also noticed to occur in the spleen, lymph nodes, and the peripheral blood of the autoimmune affected patients [9].

Reports on serologic findings in AA disease are diverse, however, knowing the state of circulating immune cells in the peripheral blood of AA patients at different stages and different subtypes of disease is very important. As it may help to expect the course and duration of disease, also to get an opportunity for developing a novel targeted therapy or at least choosing the best available one. So, we aimed in this study to assess the level of CD4, CD8 T cells, and NK cells in the peripheral blood of AA patients during the active state of the disease.

## Materials and methods

This case control study was performed on 50 patients diagnosed with active alopecia areata and 50 age and sex-matched healthy control. Patients were recruited from the outpatient clinic of Dermatology, Venereology and Andrology, Al-Azhar University, Assiut Governate in the period between October 2018 and March 2020. This study was approved by the Committee of Local Institutional Ethics of Faculty of Medicine, Al-Azhar University. Informed consent was taken from the participants after illustrating the nature of our study.

Female patients diagnosed with active alopecia areata aged 20–45 years were included in this study. We excluded patients in stable or regressive stage of the disease, patients who applied corticosteroid or other drugs for promoting hair growth in a period less than 3 months before starting the study, patients with other skin diseases rather than AA, patients with autoimmune diseases, pregnant and lactating females were also excluded from this study.

All patients were subjected to complete history taking, complete general and local examination to exclude any suspected similar diagnosis and to evaluate if there is any additional local or systemic disease contraindicating the patient from being included in the study. We collected all available data related to age, duration of the disease, past and family history of AA.

We divided patients according to their clinical type into two groups; patchy alopecia areata group (one or multiple patches of hair loss) and alopecia totalis (complete loss of the scalp hair) with alopecia universalis (complete loss of body hair) in another group.

The activity of the disease was determined clinically by hair pull test performed at the periphery of the lesion and by using dermoscope (Dermlite-4 dermatoscopy-USA) to detect the characteristic dermoscopic findings of active alopecia areata in the area of hair loss including black dots, tapering hairs and exclamation marks. There are no established criteria for determining the activity of this disease. A hair pull test with more than three hairs or a trichoscopy with a black-dot pattern and tapered/broken hairs indicates substantial disease activity. [10]

Blood samples were stained by Fluoroisothiocyanate (FITC)-conjugated CD4, phycoerythrin (PE)-conjugated CD8, phycoerythrin-cyanine5 (PC5) conjugated CD3, to detect both CD4 and CD8 lymphocytes. (FITC)-conjugated CD16, (PE)- conjugated CD56 and Allophycocyanin (APC)- conjugated CD3 were used to detect NK cells. All monoclonal antibodies were purchased from (Beckman coulter, USA). For each sample, we used isotype negative matched control antibodies to illustrate the non-specific background staining. The cells were analyzed by Navios flow cytometer (Beckman coulter, USA). CD4, CD8, CD16 and CD56 expression were evaluated and presented as a percentage of total lymphocytes. CD 4 + lymphocytes cells were defined as CD3 + CD4 + CD8− cells. CD 8 + lymphocytes cells were defined as CD3 + CD4− CD8 + cells. NK lymphocytes were defined as CD3- with either expression of CD16 and CD56 or not.

### Statistical analysis

Statistical analysis was performed using Stata/IC version 16.1 for Windows (StataCrop, LLC, College Station, TX, USA). Data are expressed in tables as mean ± standard deviation (SD), number and percentage. We used unpaired Students’t test to compare the mean of two groups of normally distributed variables while Mann Whitney U test was used for non- normally distributed data. P- value was considered significant if < 0.05.

## Results

Fifty female patients and 50 healthy female controls evaluated in this study. The mean age for the patients’ group was 26.84 ± 4.84 years ranging from 20 to 39 years and for the control group, it was 28.41 ± 6.03 years ranging from 18 to 45 years. The clinicodemographic data of AA patients are shown in Table 1.

**Table 1:** Comparison between the clinicodemographic data of alopecia areata patients

	Alopecia areata subgroups		P value
	Patchy AA	AT/AU	
Patients number	24	26	
*Age (years)*			
Mean ± SD	26.25 ± 4.36	27.38 ± 5.27	0.413
*Duration (months)*			
Mean ± SD	5.58 (2.80)	12.88 (6.08)	< 0.001
*Past history of AA (%)*			
No	22 (91.7)	19 (73.1)	0.180
Yes	2 (8.3)	7 (26.9)	
*Family history of AA (%)*			
No	21 (87.5)	21 (80.8)	0.793
Yes	3 (12.5)	5 (19.2)	
*Onset (%)*			
Sudden	23 (95.8)	26 (100.0)	0.968
Gradual	1 (4.2)	0 (0.0)	
*Course of disease (%)*			
Progressive	24 (100.0)	25 (96.2)	1.000
Transient	0 (0.0)	1 (3.8)	
*Pull test (%)*			
Negative	1 (4.2)	3 (11.5)	0.661
Positive	23 (95.8)	23 (88.5)	

AA, alopecia areata; SD, standard deviation

*P value < 0.05 was considered statistically significant

As regards the dermoscopic finding, there was no significant difference between AA subgroups as shown in Table 2.

**Table 2:** Dermoscopic findings of alopecia areata patients

	Patchy AA	AT/AU	P value
Patients number	24	26	
*Black dots (%)*			
Absent	2 (8.3)	3 (11.5)	1.000
Present	22 (91.7)	23 (88.5)	
*Exclamation mark (%)*			
Absent	4 (16.7)	4 (15.4)	1.000
Present	20 (83.3)	22 (84.6)	
*Broken hair (%)*			
Absent	5 (20.8)	7 (26.9)	0.863
Present	19 (79.2)	19 (73.1)	
*Yellow dots (%)*			
Absent	23 (95.8)	20 (76.9)	0.129
Present	1 (4.2)	6 (23.1)	
*Vellus hair (%)*			
Absent	23 (95.8)	21 (80.8)	0.229
Present	1 (4.2)	5 (19.2)	

*P value < 0.05 was considered statistically significant

Regarding the laboratory parameters of our studied population, we found that the mean white blood cells (WBCs), lymphocyte percentage, lymphocyte count, and lymphocyte-monocyte ratio were elevated in AA patients relative to the mean value of healthy control subjects as shown in Table 3.

**Table 3:** Comparison between laboratory parameters of alopecia areata patients and healthy control subjects

Laboratory parameters	Alopecia areata	Control	P value
Patients, n	50	50	
WBCs 10^3/^mm^3^	6.90 ± 1.63	6.00 ± 1.08	0.001*
Neutrophil (%)	59.77 ± 7.31	58.73 ± 4.19	0.383
Neutrophil count 10^3/^mm^3^	4.60 ± 1.21	4.29 ± 0.69	0.121
Lymphocyte (%)	38.09 ± 7.59	35.34 ± 3.56	0.022*
Lymphocytes count 10^3/^mm^3^	2.36 ± 0.55	2.14 ± 0.42	0.029*
NLR	2.06 ± 0.80	2.09 ± 0.58	0.818
Monocytes count 10^3/^mm^3^	0.20 ± 0.06	0.22 ± 0.10	0.097
Monocytes (%)	3.61 ± 1.43	4.07 ± 0.99	0.065
LMR	13.18 ± 5.44	10.99 ± 3.94	0.023*
CD4^+^ T cells (%)	33.95 ± 11.19	40.32 ± 6.42	0.001*
CD8^+^ T cells (%)	23.38 ± 7.06	31.08 ± 5.05	< 0.001*
CD4^+^/CD8^+^ ratio	1.52 ± 0.33	1.31 ± 0.22	0.001*
CD56 bright CD16^−^ (%)	1.46 ± 0.57	1.81 ± 0.79	0.012*
CD56 bright CD16 ^+^ (%)	10.10 ± 8.02	7.94 ± 4.02	0.092
CD56 ^−^ CD16 ^+^ (%)	6.62 ± 3.01	5.69 ± 2.16	0.081

WBCs, white blood cells; RBCs, red blood cells; NLR, neutrophil- lymphocytes ratio; LMR, lymphocyte-monocyte ratio; CD, cluster of differentiation

Values are expressed as means ± SD

*P value < 0.05 was considered statistically significant

**Fig. 1:**
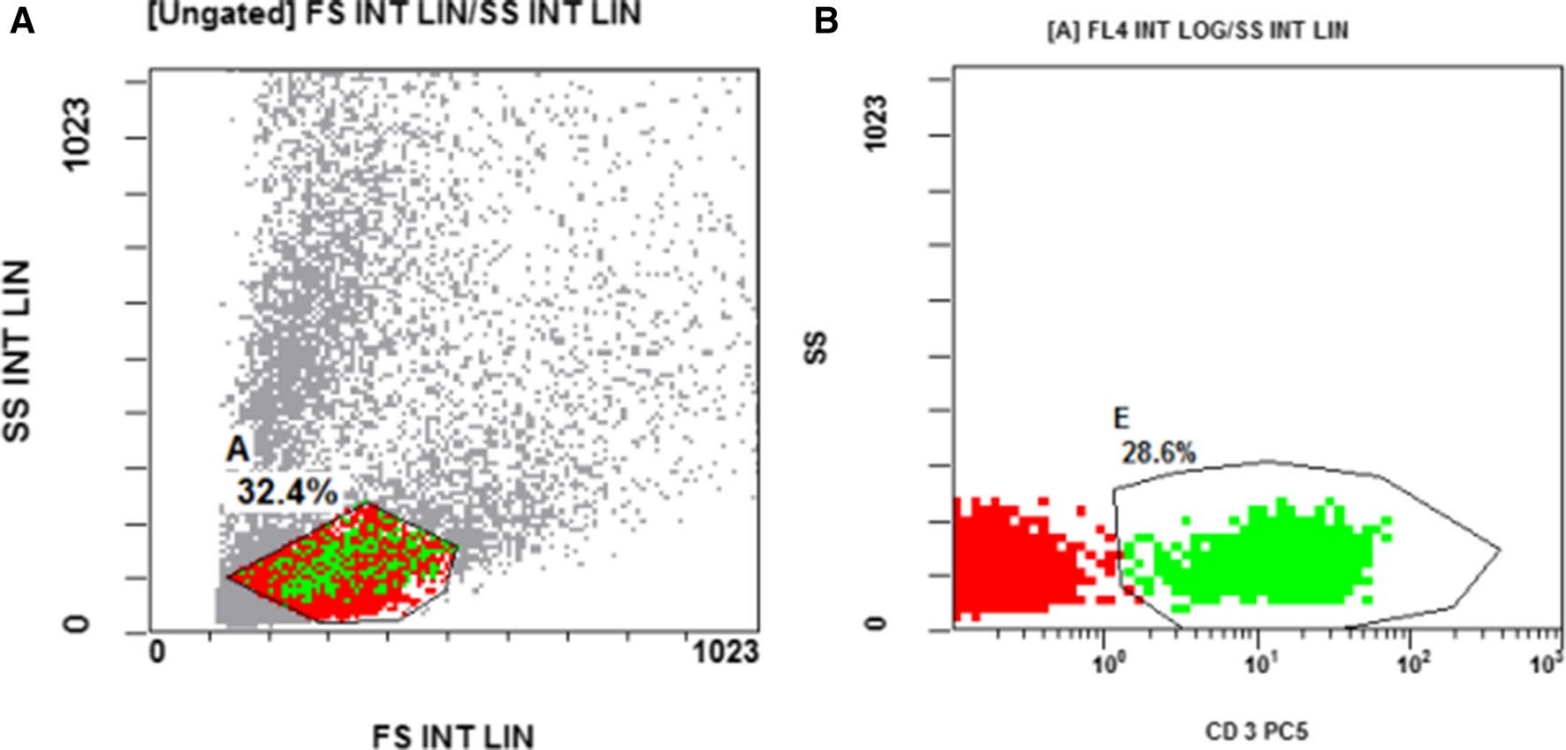
Flow cytometric detection of CD4 & CD8 lymphocytes. **a** Forward (FSC) and side scatter (SSC) dot plot was used to detect lymphocytes cells(A). **b** The expression of CD4FITC and CD8PE were assessed in region(A) to CD4 + and CD8 + lymphocytes

**Fig. 2:**
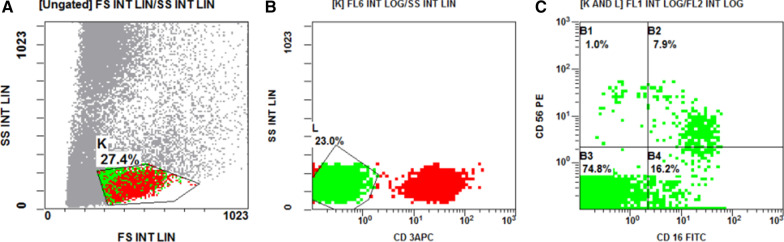
Flow cytometric detection of Natural killer cells. **a** Forward (FSC) and side scatter (SSC) dot plot was used to detect NK cells. (k). **b** CD3APC and side scatter dot plot were used to select CD3 − cells (L). **c** The expression of CD16FITC and CD56PE were assessed in CD3 − cells to detect NK cells

As regards to the level of CD4 and CD8 T cells; Fig. 1, and the level of the NK cells; Fig. 2, we found that there was a significant reduction in the level of CD4, CD8 T cells, and CD 56 bright CD16- NK cells in the peripheral blood of patients when compared to control (p = 0.001), (p < 0.001), (p = 0.012), respectively. We found a significantly higher CD4 + :CD8 + T cells ratio in the total CD3 + T-cell pool of patients’ peripheral blood compared to the control (p = 0.001) as shown in Table 3.

There was a significant positive correlation between CD4, CD8, CD56 bright CD16- % cells and disease duration (r = 0.53, p > 0.001), (r = 0.43, p = 0.002) (r = 0.45, p = 0.001), respectively as shown in Table 4.

**Table 4:** Correlation between the subtypes of lymphocytes and natural killer cells with the duration of AA disease

Immunophenotype cells	r	P value
CD4^+^ T cells (%)	0.53	< 0.001*
CD8^+^ T cells (%)	0.43	0.002*
CD4^+^/CD8^+^ ratio (%)	0.15	0.287
CD56 bright CD16^−^ (%)	0.45	0.001*
CD56 bright CD16^+^ (%)	0.03	0.824
CD56^−^ CD16^+^ (%)	0.02	0.909

r, correlation value

^*^p value < 0.05 was considered statistically significant

## Discussion

Alopecia areata is a common non-cicatricial type of hair loss; it is a site-specific autoimmune disease of hair follicles that may be patchy or diffuse or total body hair loss. Despite the growing knowledge about alopecia areata, the pathogenesis is still obscure. Many studies suggest that AA is associated with an imbalance of circulating T lymphocytes and natural killer cells [6], however, the accumulating evidence on T-cells involvement and our understanding of the mechanism is still very limited.

Literature studies are conflicting regarding the gender predominance in AA. Many studies reported that sex incidence in alopecia areata is almost equal, however, in studies done by Lundin et al., (2014); Ranawaka, (2014); Wu et al., (2013); Goh et al., (2006); Al-Ajlan et al., (2020), they reported a female predominance [11–15], while in other studies done by Alshahrani et al., (2020), Mane et al., (2011), Mahmoudi et al., (2018) they mentioned the predominance of the male gender [16–18].

The cause of observed specific sex towards the occurrence of autoimmune diseases is still unclear, however proposed hypothesis return that to the differences in the activation of androgen and estrogen receptor of the immune cell, another theory suggests that X chromosome may have a role in the immune response [11]. To avoid the potential influence of gender on our result, we restricted the studied population to female gender.

To date, few studies have been focused on the hematological and inflammatory parameters of AA and disease activity.

WBCs and lymphocyte count were significantly higher in AA patients as compared to the healthy subjects, however, neutrophil and monocyte count did not show a significant difference between both groups, and this comes in accordance with the results reported by İslamoğlu and Demirbaş, (2020) [19]. This can be explained by studies done by Herbst et al., (2006) and Seleit et al., (2018), as they mentioned that the peripheral blood leukocytes from AA patients are relatively resistant to apoptosis, which might be due to gene polymorphisms of Fas Ligand (FasL) resulting in a decrease in its expression and programmed cell death dysfunction [20, 21].

CD4^+^T helper cells along with CD8^+^T cytotoxic cells make up the majority of T-lymphocytes. CD4 T cells after being activated and differentiated into different subtypes play a critical role in mediating immune response through activation of other cells including B cells, CD8 T cells, mast cells, and macrophages [22]. CD4 + T cells serve as costimulators because optimum hair loss induction necessitates the precence of both CD4 + and CD8 + T cells [23].

In our study, CD4 T and CD8 T cells were less frequent in peripheral blood of patients compared to healthy control in line with the increase in CD4:CD8 T cell ratio agreeing with a study done by Lee et al., (1996) [24]. This is also in agreement with the study done by Lutz et al., (1988) as they reported a highly significant reduction of CD8 T cells in patients when compared to control, however, CD4 T cells reduction in patients did not show a significant difference [25].

The reduction in CD4 + and CD8 + T cells in our study can be explained by the work of Zöller et al., (2004) as they reported that peripheral blood of patients with active AA showed a higher percentage of T regulatory cells (T reg) that muchly suppress the proliferative activity of CD4 + and CD8 + T cells when compared with healthy control or patients with stable or regressive AA [9].

Also, in a study done by Kubo et al., (2017) they indicated that patients with short disease duration associated with a higher proportion of Treg cells in the peripheral blood, which suppresses the disease activity and proliferation of CD4 + , CD8 + T cells, and NK cells at the early phase. However, with prolonged disease duration, the level of regulatory T cells declines in the peripheral blood leading to the disease progression [26]. Thus, this helped us to explain the positive correlation result between the duration of the disease and the proportion of CD4 + and CD8 + T-cells in the peripheral blood of our studied population, also explained why alopecia totalis and universalis with the longest disease duration associated with a higher proportion of CD4 + , CD8 + T-cells in the peripheral blood when compared to alopecia unilocularis.

Then, if there is a reduction in the proportion of CD4 + and CD8 + T-cells in the peripheral blood of AA patients, why the proportion of those cells are increased around the immune privilege of hair follicles leading to their destruction? Interestingly, this can be explained through the study done by Hamed et al., (2019) as they found that there is a higher proportion of CD4 + and CD8 + T cells around hair follicles are due to the decline in the T regulatory cells proportion by 90% in areas of the skin with active AA when compared to the normal skin despite being 40% higher in peripheral blood of AA patients when compared to controls [27]. In addition, Lima et al., (2015) and his colleagues found that recruitment of the immune cells to the lymphoid organs and homing to the lesional site is mediated by the interaction of homeostatic chemokines on locally resident cells, with matched chemokine receptors (CKR), so CCR5/CXCR3 + expression on CD4 T cells and CCR5/CXCR3 + of CD8 T cells play a role in homing of these cells to inflamed tissues [28]. So, the reduction in the proportion of the circulating CD4 + and CD8 + T cells likely due to the migration, trafficking and accumulation of those cells to the site of the lesion. This hypothesis is also supported by Dai and his colleague in 2016, as they found that in mice with active AA, CD8 + T cells migrated to the site of the lesion depending on CXCR3 signaling and blocking these receptors resulting in inhibition of T cell migration into lesional site and preventing the development and progression of AA [29].

In fact, the disturbance in immune cells not only affecting CD4 + and CD8 + T-cells, but also the expansion or reduction of NK cells and their cytokines are noticed in multiple autoimmune diseases either in the peripheral blood or at the site of the lesion.

To the best of our knowledge, this is the first description of the proportion of NK cells based on CD56 and CD16 expression in the peripheral blood of AA patients during the active stage of AA disease.

NK cells are considered the third main lymphocytic compartment after B and T cells and with different phenotypic and functional. NK are subdivided according to their surface markers CD56 and CD16 into many subtypes. Normally, in the healthy subjects’ peripheral blood CD56dim CD16bright represent approximately 90% of all NK cells. However, CD56bright subset represents only 10% of NK cells [30].

In this study, we investigated the frequency and cell surface phenotype of NK cells in the peripheral blood of AA patients and their relation to the disease activity and alopecia areata subtypes. Interestingly, we found a significant reduction in the level of CD56 bright CD16- NK cells in the peripheral blood of AA patients when compared to healthy control.

Our results are matched with data reported by Lutz et al., (1988) as they declared that Leu-11a + CD16 (Fc IgG receptor) of NK cells are reduced in the patients’ circulation in comparison to control [25].

Lima and his colleagues discovered that NK-cells are able to circulate in the blood and reach the lesional site by using CKR, and adhesion molecules. CXCR3/CCR5 on CD56bright NK-cells permit these cells to migrate into the affected tissue [28], so we can explain the reduction in the number of peripheral NK cells that may be due to chemokine-dependent NK cell recruitment from peripheral blood to the site of the lesion.

Ito et al. in 2008 reported that CD56bright NK cells are accumulated around the hair follicle of AA patients [31], also, it was reported that CD56bright NK cells are accumulated in the skin lesions of psoriatic patients and the synovium of rheumatoid arthritis patients [32–34]. These observations support the hypothesis that decreased NK cells in the peripheral blood of patients with autoimmune disorders may reflect the trafficking of NK cells to affected tissues [35].

Now, we have another question regarding our results, why there is a decline only in the proportion of CD56bright CD16- NK-cells, while the proportion of other subpopulations showed no difference in comparison to the control group.

This could be explained by the work of Lima et al., (2015) as they reported that NK-cell subsets have a different CKR, so it can circulate in the blood or migrate into inflamed tissues, with different immune cells, and in several different circumstances, in response to constitutive and inflammatory chemokines [28].

The restriction only on patients with active stage of the disease without comparing patients in different stages of diseases was considered as a limitation of our study (Additional file 1).

## Conclusion

To sum up our results, this work demonstrated that the active stage of AA disease is associated with a reduction in the proportion of circulating lymphocytes and NK cells in the affected patients. Our findings also provide a novel insight into the pathogenic mechanism behind the observed reduction in the immune cells. Immunomodulating agent having the ability to block the migration of activated immune cells from the peripheral blood to the site of the lesion could be effective in normalizing the disturbance of those immune cells during the early active stage of this disease. However, further studies are needed to clarify the clinical importance of our speculations.

## Supplementary Information

**Additional file 1.** Row data of tables.

## Data Availability

The datasets used and/or analysed during the current study are available from the corresponding author on reasonable request.
